# Optic Neuritis and Vertebral Osteomyelitis: An Uncommon Presentation of Cat-Scratch Disease

**DOI:** 10.7759/cureus.52284

**Published:** 2024-01-15

**Authors:** António Carujo, André Santos Silva, Fábio Videira Santos, Maria João Furtado, António Ludgero Vasconcelos

**Affiliations:** 1 Infectious Diseases Department, Santo António University Hospital, Porto, PRT; 2 Abel Salazar Biomedical Sciences Institute, University of Porto, Porto, PRT; 3 Ophthalmology Department, Santo António University Hospital, Porto, PRT

**Keywords:** bartonella, spondylodiscitis, osteomyelitis, optic neuritis, cat-scratch disease

## Abstract

In cat-scratch disease (CSD), hematogenous spread may result in atypical presentations. Ocular manifestations develop in a minority of patients, with treatment being important in reducing long-term visual sequelae. Bone infection is rare. We present the case of a 52-year-old woman, with close contact with cats, reporting acute unilateral blurred vision and presenting papilledema, optic disc pallor, and peripapillary hemorrhage. Etiologic study of optic neuritis revealed an elevated positive *Bartonella* IgG; hence, treatment for CSD with doxycycline plus rifampin and corticosteroids was started. Concomitant lumbar pain of increasing intensity warranted magnetic resonance imaging, which revealed L3-L4 vertebral osteomyelitis with spondylodiscitis. Given the temporal link with CSD diagnosis and the significant clinical improvement since its treatment was started, an etiologic link was presumed and antibiotics were prolonged. This case stands out for the presence of distinct atypical CSD manifestations in the same patient. Further studies are needed to determine the optimal treatment for rare manifestations, particularly bone infection.

## Introduction

Cat-scratch disease (CSD) results mainly from a scratch or bite from an infected cat [1]. *Bartonella henselae *is the etiologic agent in most cases and cats serve as the natural reservoir [1].

CSD is typically characterized by a localized cutaneous lesion with regional lymphadenopathy near the site of organism inoculation [1,2]. Hematogenous spread may result in atypical manifestations, including visceral organ, ocular, neurologic, and musculoskeletal disease, which are more likely in the elderly [1-3]. Ocular manifestations, including optic neuritis, develop in a minority of patients with CSD and generally have a good long-term prognosis if treated timely [1,4,5]. Long-term visual deficits may develop in patients with macular exudates forming the macular star, the typical presentation of neuroretinitis, where *B. henselae* is among the most common infectious causes [4,6]. Musculoskeletal manifestations include tendinitis, arthritis, and osteomyelitis [7]. Bone infection is rarely reported [7,8].

Antimicrobial therapy should be administered to all patients with CSD, including those with localized disease that generally have a self-limited illness, as treatment can shorten the duration of symptoms and prevent complications [2,9]. In the presence of ocular manifestations, treatment may also reduce the risk of long-term visual sequelae [9].

## Case presentation

**Figure 1:**
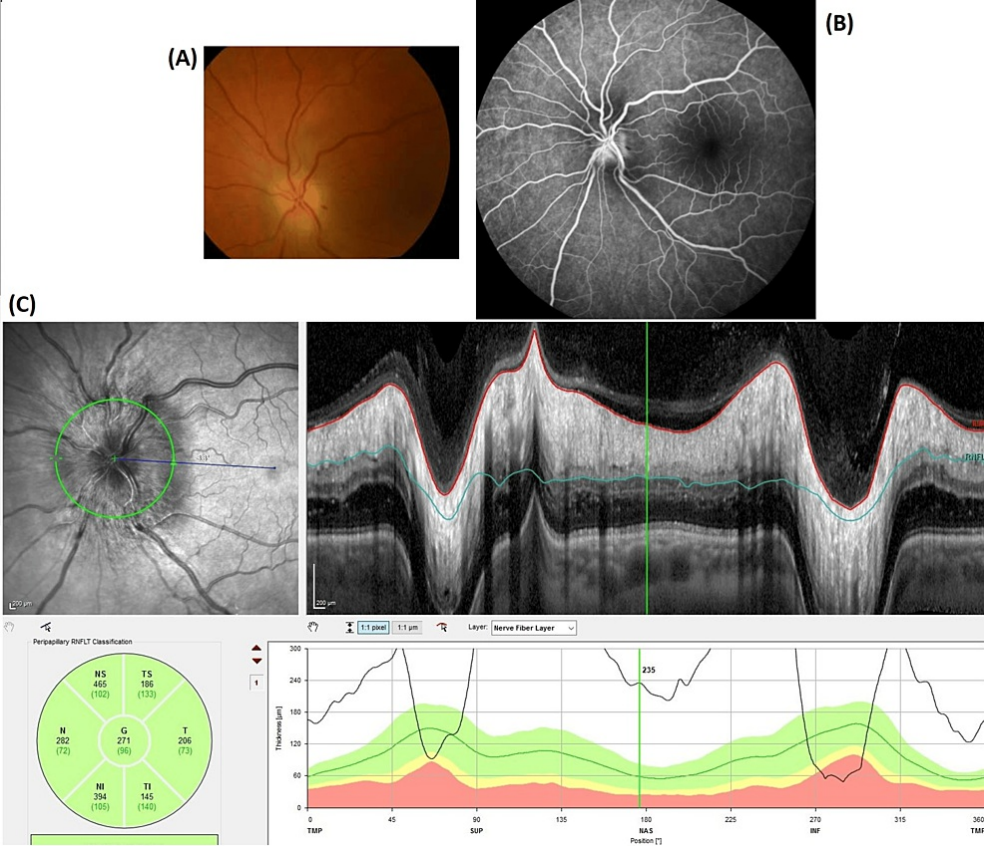
(A) Fundus photo. (B) Fluorescein angiography. (C) Optic disc optical coherence tomography. Optic disc leakage was noted in the setting of optic neuritis.

**Figure 2:**
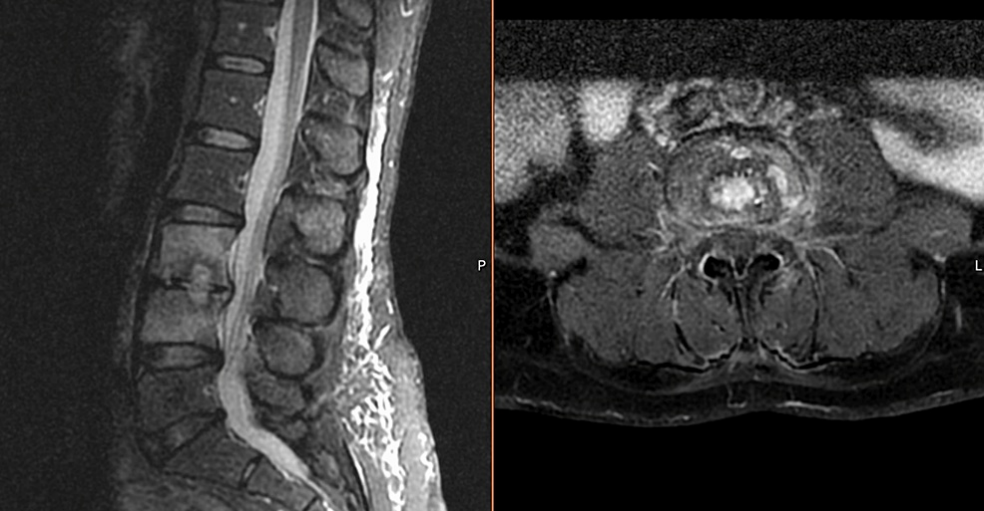
L3-L4 vertebral osteomyelitis with spondylodiscitis on magnetic resonance imaging.

We present the case of a 52-year-old woman with no clinical background, living in rural northern Portugal with close animal contact, including cats. Due to five days of blurred vision in the left eye, the patient attended the Emergency Department. She reported no fever, headache, eyeache, or cutaneous lesions. Ophthalmologic examination revealed unilateral visual loss characterized as 8/10, with papilledema, optic disc pallor, and peripapillary hemorrhage (Figure 1). Additionally, she reported a non-radiating lumbar pain of increasing intensity in the past three weeks. Physical examination presented no cutaneous signs of trauma or infection on inspection but intense pain on palpation and percussion of the lumbar spinous processes, with no associated neurological deficits. These findings prompted a magnetic resonance imaging (MRI) that revealed L3-L4 vertebral osteomyelitis (Figure 2).

Etiologic study of optic neuritis included venereal disease research laboratory (VDRL) for syphilis, *Borrelia *serology, interferon-gamma release assay for tuberculosis, and, considering the epidemiologic setting, *Bartonella* serology. Human immunodeficiency virus infection was excluded, and a basic immunology study revealed no immunocompromise. Polymerase chain reaction (PCR) for *Bartonella* performed on peripheral blood was negative, but serology revealed an IgG of 1:128. IgM was negative, but testing was performed over a month after symptom onset. Because IgG titer alone can confirm CSD, treatment with doxycycline (100 mg bid) plus rifampin (300 mg bid) was started. In addition, a six-week course of oral steroids was administered (prednisolone 1 mg/kg for two weeks, with subsequent gradual taper). A lumbar puncture was performed, with unremarkable cerebrospinal fluid findings including negative PCR for *Bartonella*, VDRL, and *Borrelia *IgG.

Regarding vertebral osteomyelitis with spondylodiscitis, a biopsy was performed. However, despite being image-guided, the histopathology suggested an inaccurate collection of intervertebral disc with no vertebral bone sample. This was further confirmed by sterile bacteriological and mycobacterial cultures and negative PCR for *Bartonella*, *Brucella*, and *Mycobacterium tuberculosis*. Blood cultures were also sterile, and *Brucella* and *Coxiella* serologies were negative. Given the temporal link with CSD diagnosis and the significant clinical improvement with its treatment, an etiologic link with CSD was presumed and antibiotics were prolonged for three months.

**Figure 3:**
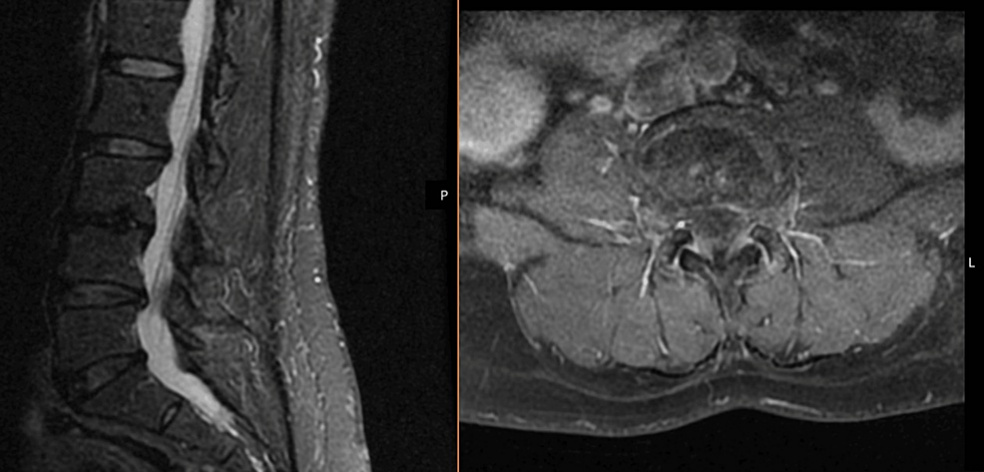
Inflammatory resolution in comparative lumbar magnetic resonance imaging after three months of treatment.

**Figure 4:**
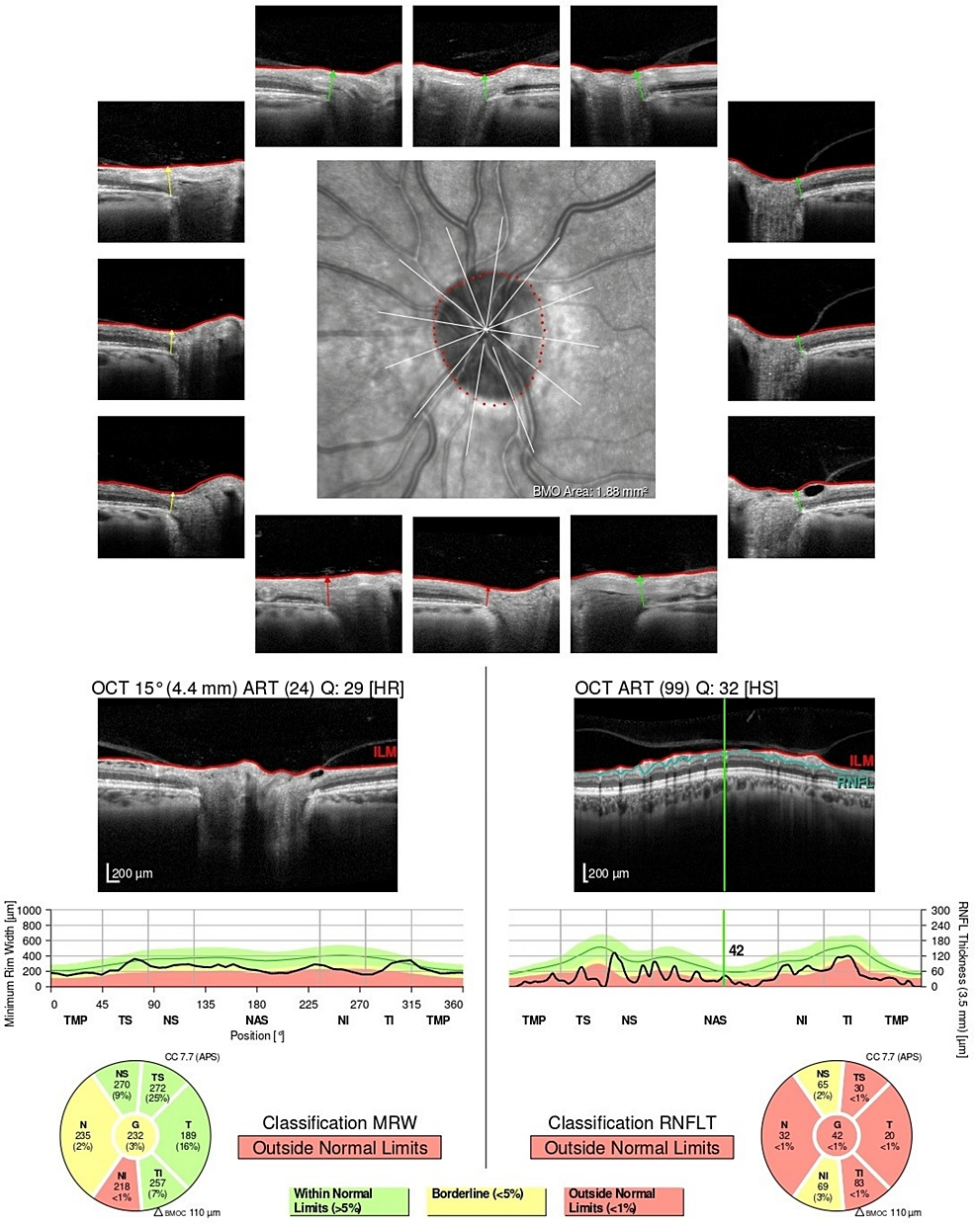
Optic disc optical coherence tomography showing loss of peripapillary retinal nerve fiber layer thickness.

Evaluation of the patient after completion of the antibiotic treatment revealed significant symptomatic improvement, inflammatory resolution in comparative lumbar MRI (Figure 3), and negative follow-up *Bartonella* serology with an IgG of 1:64. Optical coherence tomography demonstrated sequelae with loss of peripapillary retinal nerve fiber layer thickness (Figure 4).

## Discussion

Serologic testing for Bartonella presents poor overall sensitivity and specificity [10]. It should be performed to confirm CSD in the presence of suggestive epidemiologic and clinical features but, when negative, does not rule out infection and should not delay therapy [1]. IgM production is brief, but IgG titers alone greater than 1:64 represent possible active or recent infection [1,11]. Repeat serologic testing should be performed in 14 days [1].

Isolation from blood and tissue samples remains very difficult as *B. henselae* is a slow-growing bacteria that requires specific laboratory conditions for optimal growth, including a prolonged incubation period [1]. Therefore, when biopsy samples are obtained, testing includes histology and PCR [1]. PCR tests have high specificity, further allowing distinction between *Bartonella* species [12]. However, the timing of the biopsy greatly influences its results, as it is more likely to be positive if performed in the first six weeks of infection [13]. Although PCR in blood samples should not be performed routinely given low sensitivity, it may be reasonable in those with atypical manifestations [1].

CSD-related bone infection is rare, with a literature review reporting only 47 cases, affecting most commonly the spine (n = 27), of which only four had discitis [8]. Cases were defined by bone infection during CSD or when the causative agent was demonstrated in the bone [8]. Bone biopsy was performed in solely 13 patients, in whom PCR was positive in only five, and histology suggestive in two [8]. The authors suggested avoiding invasive demonstration of *Bartonella* in the bone, except when other important etiologies are suspected [8]. In our case, the etiologic study of vertebral osteomyelitis was further complicated by the inaccurate tissue collection. Given the temporal link with CSD diagnosis and the significant clinical improvement with treatment, an etiologic link with CSD was presumed. In the literature, we could not find any patient with both CSD-related bone and ocular manifestations, making this case truly unique.

For those with disseminated disease, therapy combination with rifampin is advised [9]. In the case of ocular disease, doxycycline is preferred in association with rifampin, with treatment extended from four to six weeks and close monitoring by an ophthalmologist [6,9]. In vitro susceptibility testing often does not correlate with the clinical response and, therefore, should not be considered in the choice of antibiotics [14]. In addition, adjunctive corticosteroids are warranted in severe or refractory cases, as well as in patients with ocular manifestations, as visual improvement is significantly superior [5,9]. Due to the additional musculoskeletal involvement, we further prolonged antibiotic treatment in our patient for three months. A literature review has presented the therapeutic regimens for bone infection that have been associated with cure, consisting of various antibiotics used sequentially or in combination, for different durations [8]. There are no evidence-based guidelines due to the lack of comparative data between regimens [8].

## Conclusions

This case stands out for the presence of atypical CSD manifestations in the same patient: optic neuritis and osteomyelitis with spondylodiscitis. Diagnostic tests should be performed to confirm a clinical impression of CSD but, when negative, should not delay therapy. Although treatment regimens for ocular manifestations are well established, further studies are needed to determine the optimal management strategy for rarer manifestations such as bone infection.
